# Significance of serum sestrin2 as a biomarker of severity and functional outcome in acute intracerebral hemorrhage: a prospective observational longitudinal study

**DOI:** 10.1186/s12883-023-03470-6

**Published:** 2023-11-29

**Authors:** Xianghong Dou, Wensheng Dong, Yanmei Gu, Tingting Zhang, Jianhua Zhang

**Affiliations:** 1 Department of Neurology, Donghai County People’s Hospital, Lianyungang, 222300 Jiangsu China; 2 Department of Neurosurgery, The Second People’s Hospital of Lianyungang, Lianyungang, 222000 Jiangsu China; 3 Department of Neurology, Ganyu District Traditional Chinese Medicine Hospital of Lianyungang, Lianyungang, 222100 Jiangsu China

**Keywords:** Sestrin2, Intracerebral hemorrhage, Early neurologic deterioration, Prognosis, Severity, Biomarkers

## Abstract

**Background:**

Sestrin2 is a highly conserved stress-inducible protein with neuroprotective properties. Herein, we investigated the prognostic significance of serum sestrin2 in human intracerebral hemorrhage (ICH).

**Methods:**

In this prospective observational longitudinal study, we enrolled 126 patients with supratentorial ICH as cases together with 126 healthy individuals as controls. Severity indicators were National Institutes of Health Stroke Scale (NIHSS) and hematoma volume. Prognostic parameters were early neurologic deterioration (END) and post-stroke 6-month poor prognosis [modified Rankin Scale (mRS) scores of 3–6]. Multivariate analysis was performed to assess relations of serum sestrin2 levels to severity and prognosis.

**Results:**

Patients had statistically significantly higher serum sestrin2 levels than controls. Serum sestrin2 levels of patients were independently correlated with NIHSS scores and hematoma volume, as well as were substantially elevated in order of mRS scores from 0 to 6. Serum sestrin2 was identified as an independent predictor of END and poor prognosis. Based on the receiver operating characteristic curve, serum sestrin2 had a similar predictive ability for END and poor prognosis, as compared to NIHSS scores and hematoma volume. Prediction models of END and poor prognosis, in which serum sestrin2, NIHSS scores and hematoma volume were integrated, were visually described via nomogram, were reliable and stable under calibration curve and were of clinical benefit using decision curve analysis. Also, prediction model of poor prognosis showed dramatically higher discriminatory efficiency than any of NIHSS scores, hematoma volume and serum sestrin2.

**Conclusion:**

Serum sestrin2 levels, which are obviously increased following acute ICH, are independently related to illness severity and poor clinical outcomes, substantializing serum sestrin2 as a clinically valuable prognostic biomarker of ICH.

## Introduction

Intracerebral hemorrhage (ICH) is an illness caused by accumulation of bleedings within brain parenchyma, thereby resulting in neuronal damage, neurological impairments and even death of patients [1]. ICH is well-known to induce secondary brain injury, which involve a series of molecular mechanisms, including inflammation, oxidative reaction and neuronal death [2]. Up to data, this disease is devoid of effective therapeutic modalities and therefore its clinical outcome is unsatisfactory [3]. Early neurologic deterioration (END) is frequently observed in patients with ICH and its appearance often increases risk of poor prognosis after ICH [4]. The prognostic prediction and severity estimation of ICH is currently based on clinical and radiological evaluations [5]. The clinical utilities of National Institutes of Health Stroke Scale (NIHSS) and hematoma volume in prognostication of ICH are believably acceptable in daily practice [6, 7]. Interestingly, molecular biomarkers of ICH have been extended to measurement in easily obtainable peripheral blood and their prognostic potentials have been paid more attentions in recent times [8, 9].

Sestrin2 belongs to the sestrin family and is a highly conserved stress-inducible protein [10]. Sestrin2 may take possession of protective effects via scavenging free radicals and reducing apoptosis, and its expressions can be induced by numerous environmental stresses such as inflammation, oxidative stress, ischemia and hypoxia [11, 12]. After acute brain injury caused by experimental traumatic brain injury, ischemic stroke or subarachnoid hemorrhage, sestrin2 expressions by neurons and microglia were substantially elevated and overexpression of sestrin2 could significantly reduce oxidative injury, alleviate neuroinflammatory insults, attenuate blood-brain barrier disruption, depress neuronal apoptosis and thereby improve neurologic function [13–15]. Thus, sestrin2 may be neuroprotective. Intriguingly, serum sestrin2 levels were substantially higher in patients with Parkinson’s disease than in controls [16]. Also, in patients sustaining Alzheimer’s disease or mild cognitive impairment, a significant elevation of serum sestrin2 levels was found, as compared to elderly controls [17]. Therefore, serum sestrin2 may be a biomarker for reflecting oxidative damage. Nonetheless, it is unknown whether serum sestrin2 levels are altered in humans after acute brain injury. Here, we investigated the change of serum sestrin2 levels in a cohort of ICH patients and further determined the prognostic role of serum sestrin2 in human ICH.

## Materials and methods

### Participant enrollment

In this prospective observational study from April 2018 to September 2021, patients with primary ICH, who were admitted to the Second People’s Hospital of Lianyungang, constituted study cases. The inclusion criteria for the case group were shown below: (1) voluntary entry into study; (2) age of at least eighteen years; (3) first-ever stroke; (4) supratentorial ICH; (5) bleedings conservatively treated; and (6) hospital admission time equal to or less than 24 h since symptom onset. The exclusion criteria for the case group were delineated as follows: (1) some previous neurologic diseases, such as stroke, intracranial tumors, Parkinson’s disease, Alzheimer’s disease and multiple sclerosis; (2) some coexisting systemic or severe diseases, such as malignancies, autoimmune system diseases, acute myocardial disease, liver cirrhosis, uremia and other organ function failure; and (3) some specific conditions, such as loss to follow-up, incomplete data and unavailable samples. A group of healthy volunteers were recruited as controls. The protocol of the current study fitted the guidelines of the Declaration of Helsinki and its later amendments. This study was authorized by the ethics committee at the Second People’s Hospital of Lianyungang and written informed consent was acquired from patients’ relatives or controls themselves.

### Data collection

Upon arrival at emergency center, patients’ relatives were inquired about demographic information, including age, gender, body weight and height. Body mass index (BMI) was calculated based on the equation as follows: body weight (kg) divided by height squared (m^2^). We also registered some vascular risk factors, which included adverse life habits (namely, cigarette smoking and alcohol drinking) and specific chronic comorbidities (i.e., hypertension, diabetes mellitus, hyperlipidemia and coronary heart disease). Medication histories were carefully investigated and thereafter statin, anticoagulant, antiplatelet, antihypertensive, hypoglycemic and insulin uses were recorded. Admission arterial blood pressures were measured using non-invasive technique. Admission time was referred to as period between onset of symptom and hospital admission. Sampling time was designated as period from symptom onset to admission into hospital. Neurologic deficit was assessed using NIHSS. Radiological parameters of supratentorial hematoma included size calculated in 0.5×a×b×c [18], locations (lobar and deep) and subarachnoidal or intraventricular extension of hematoma. If increase in NIHSS score of patients was 4 or greater within post-admission 24 h or patients were deceased, END was considered [19]. The modified Rankin scale (mRS) scores varied from 0 to 6, with higher score indicating poorer neurologic dysfunction and score 6 denoting death [20]. Six-month follow-up was implemented and scores 3–6 was accepted as a poor prognosis [21].

### Measurements of serum sestrin2

Five ml of antecubital venous blood, which was drawn from ICH patients and control subjects, was immediately put into gel-containing biochemistry tube. After centrifugation for 20 min at 3000 ×g, supernatants were isolated. Acquired serum was transferred into Eppendorf tubes and preserved at a − 80 ◦C freezer until further detection. Serum sestrin2 levels were gauged using enzyme-linked immunosorbent assay (ELISA) in compliance with the manufacturer’s instructions. ELISA kits were purchased from Bioassay Technology Laboratory (Shanghai, China; catalog number, E3437Hu), with detection sensitivity of 0.01 ng/ml and detection range of 0.05-15 ng/ml. Its intra- and inter- assay coefficients of variation were less than 8% and 10%, respectively. All measurements were in duplicate done by an experienced technician, who was not familiar with clinical data.

### Statistical analysis

The SPSS 23.0 software (SPSS Inc., Chicago, IL, USA) and R software (version 3.5.1; https://www.r-project.org) were operated for statistical analysis. Using the Kolmogorov Smirnov test, age, body mass index, and arterial blood pressure were verified to be normally distributed and therefore were reported as mean values and standard deviations (SDs); and the other continuous variables were non-normally distributed and consequently were summarized as medians and percentiles with lower-upper quartiles. As for comparisons of continuous data between two groups, the independent-samples Student’s t-test and Mann-Whitney U test were applied for parametric and non-parametric comparisons respectively. The Kruskal-Wallis test was employed to compare non-normally distributed continuous variables among multiple groups. Categorial data were reported in form of counts (percentages) and their intergroup comparisons were completed using the chi-square test or exact Fisher’s test as appropriate. Because serum sestrin2 levels were distributed non-normally, its correlation with other variables were assessed using Spearman’s correlation coefficient test. Afterwards, the significantly correlated variables in univariate analysis were forced into the multiple linear regression model to ascertain independent variables and the correlations were reported as beta (β) and the corresponding 95% confidence interval (CI). Univariate analyses were done to reveal some variables with significant differences between patients with END and those not presenting with END, as well as between patients with poor prognosis (mRS scores 3–6) and those with good prognosis (mRS scores 0–2); and then those significant variables were incorporated into the binary logistic regression models to discern independent predictors of END and poor prognosis, and the associations were reported as odds ratio (OR) values. Two nomograms were built to visually delineate independent predictors of END and poor prognosis. Prediction models were assessed using calibration curve and decision curve. Under the receiver operating characteristic (ROC) curve, area under ROC curve (AUC) was generated for evaluating discriminatory efficiency and using Youden method, a best threshold value was yielded for prognostic assessment. The two-sided *P* < 0.05 denotes statistically significant differences.

## Results

### Participants’ selection and characteristics

According to the prespecified inclusion criteria, an aggregate of 160 patients with primary supratentorial ICH obtained an initial assessment, and based on the predefined exclusion criteria, 34 patients were removed from this study because of presence of previous neurologic diseases (14 cases), coexisting systemic or severe diseases (15 cases), and some specific conditions (5 cases). At last, 126 patients were eligible for further investigation. Simultaneously, 126 healthy volunteers were recruited as controls. There were no statistically significant differences in terms of age, BMI, as well as gender, smoker and drinker percentages between patients and controls (all *P* > 0.05; Table 1).

**Table 1:** Basic characteristics between healthy controls and patients with acute intracerebral hemorrhage

Variables	Patients	Controls	*P* value
Number	126	126	
Age (years)	61.9 ± 10.2	60.6 ± 14.1	0.303
Gender (male/female)	69/57	60/66	0.257
Body mass index (kg/m^2^)	24.2 ± 3.4	23.8 ± 3.3	0.094
Cigarette smoking	47 (37.3%)	46 (36.5%)	0.896
Alcohol drinking	49 (38.9%)	45 (35.7%)	0.602

Data were reported as count (percentage) or mean ± standard deviation based on datum patterns. Statistical methods included the Chi-square test and Student’s 𝑡-test

This group of patients were inflicted with several chronic diseases, such as hypertension (75 cases), diabetes mellitus (31 cases), hyperlipidemia (40 cases) and coronary heart disease (10 cases). As for medications, 33 patients used statins; 7, anticoagulants; 18, antiplatelet agents; 67, antihypertensive drugs; 25, hypoglycemic drugs or insulin. Patients were admitted into hospital from 0.3 to 23.8 h (median, 9.1 h; percentiles 25th-75th, 5.6–13.8 h) following onset of stroke. Their blood was drawn from 0.8 to 25.4 h (median, 10.8 h; percentiles 25th-75th, 6.6–15.5 h) following ICH. As regards non-invasively measured blood pressure, systolic arterial pressure ranged from 95 to 209 mmHg (mean, 142.9 mmHg; SD, 23.0 mmHg) and diastolic arterial pressure, from 67 to 111 mmHg (mean, 85.7 mmHg; SD, 9.8 mmHg). With regard to hematoma features, there were 33 cerebral lobar hematomas and 96 deeply-located hematomas, a total of 31 and 11 patients presented with hematomas extended into intraventricular and subarachnoid space respectively and bleeding size ranged from 2 to 42 ml (median, 13 ml; lower-upper quartiles, 8–21 ml). Concerning neurologic assessment, NIHSS scores varied from 0 to 17 (median, 10; lower-upper quartiles, 7–12).

### Change of serum sestrin2 levels after Stroke and its relation to Stroke severity

**Fig. 1:**
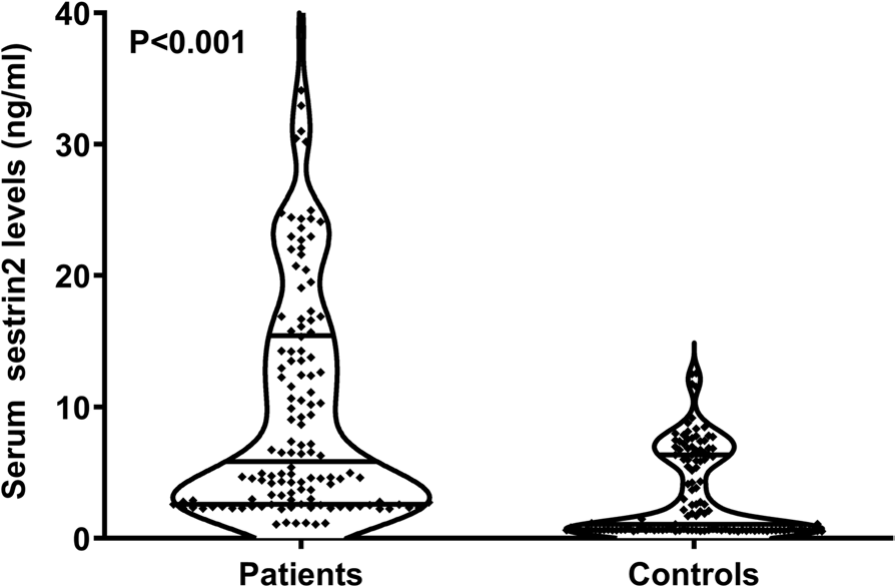
Violin plot showing change of serum sestrin2 levels after acute intracerebral hemorrhage.  Serum sestrin2 levels were dramatically higher in patients with acute intracerebral hemorrhage than in controls ( *P*  < 0.001)

**Fig. 2:**
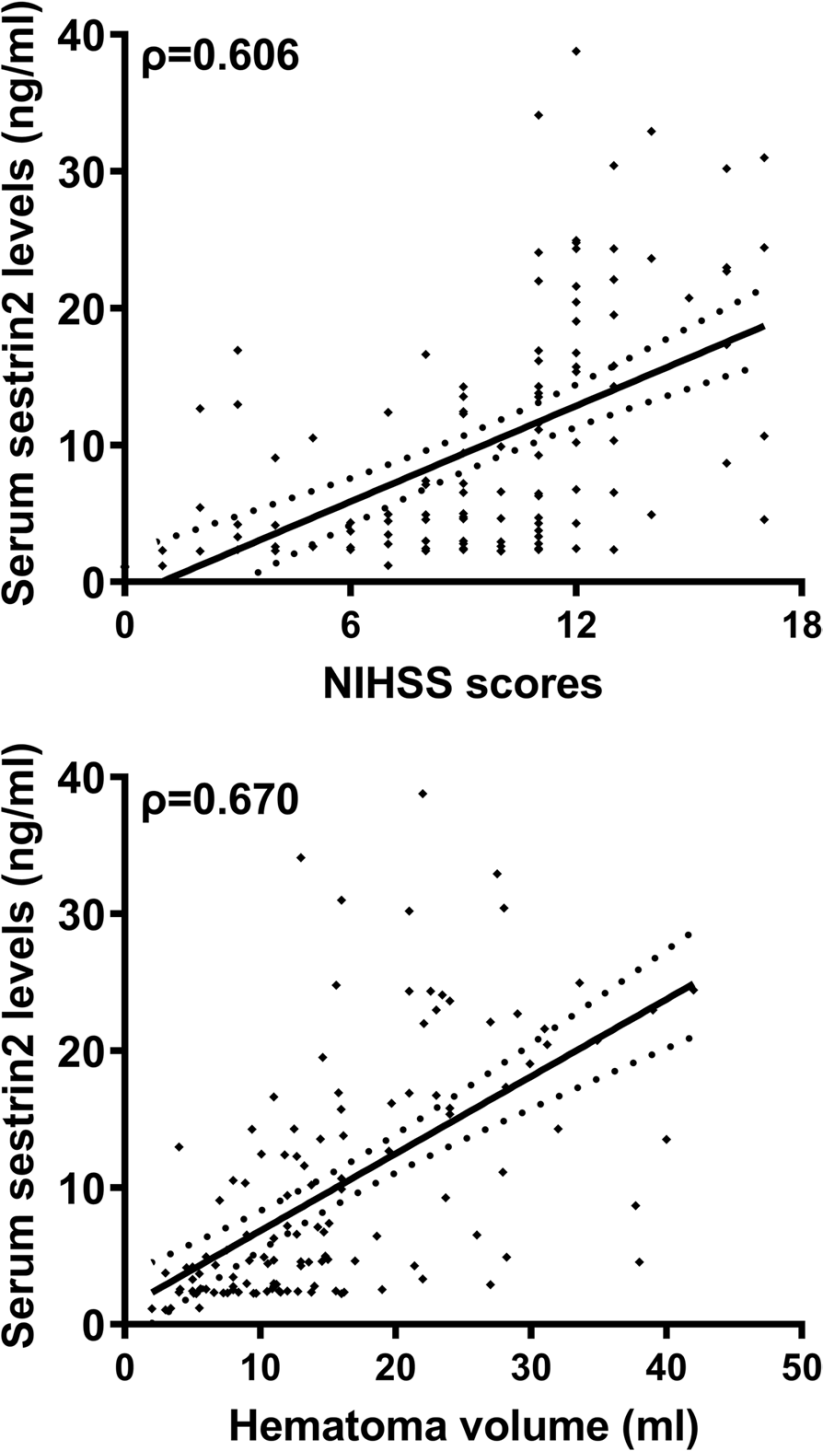
Scatter plot displaying relation of serum sestrin2 levels to stroke severity of acute intracerebral hemorrhage.  Serum sestrin2 levels had significantly positive correlation with National Institutes of Health Stroke Scale scores and hematoma volume of patients with acute intracerebral hemorrhage (both *P*  < 0.001).  NIHSS indicates National Institutes of Health Stroke Scale

**Fig. 3:**
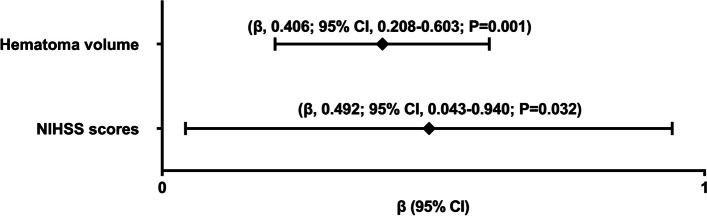
Forest plot showing multivariate linear regression results of serum sestrin2 levels after acute intracerebral hemorrhage.  Serum sestrin2 levels were independently correlated with National Institutes of Health Stroke Scale scores and hematoma volume of patients with acute intracerebral hemorrhage (both *P*  < 0.05).  NIHSS indicates National Institutes of Health Stroke Scale; β, beta; 95% CI, 95% confidence interval

As delineated in Fig. 1, serum sestrin2 levels were statistically significantly higher in patients with ICH than controls (*P* < 0.001). For the sake of exploring whether serum sestrin2 levels were pertinent to hemorrhagic severity, NIHSS scores and hematoma volume were selected as the two severity indicators and bivariate correlations were implemented using the Spearman test. In Fig. 2, there was a significantly positive correlation between serum sestrin2 levels and NIHSS scores (*P* < 0.001), as well as between serum sestrin2 levels and hematoma volumes (*P* < 0.001). In Table 2, besides NIHSS scores and hematoma volume, intraventricular hemorrhage and blood glucose levels were highly positively related to serum sestrin2 levels (both *P* < 0.05). Next, the aforementioned four significantly correlative variables were entered into the multivariate linear regression model, and it was proved that NIHSS scores and hematoma volumes were independently correlated with serum sestrin2 levels (both *P* < 0.05; Fig. 3).

**Table 2:** Factors correlated with serum sestrin2 levels after acute intracerebral hemorrhage

Variables	ρ	*P *value
Age (years)	-0.120	0.182
Gender (male/female)	-0.008	0.932
Body mass index (kg/m^2^)	-0.007	0.941
Hypertension	0.044	0.623
Diabetes mellitus	0.077	0.390
Hyperlipidemia	0.106	0.238
Coronary heart disease	0.168	0.060
Cigarette smoking	-0.013	0.888
Alcohol drinking	-0.039	0.667
Statin use	0.051	0.568
Anticoagulant use	0.084	0.348
Antiplatelet use	-0.026	0.776
Antihypertensive use	-0.035	0.696
Hypoglycemic use	0.013	0.886
Admission time (h)	0.085	0.341
Sampling time (h)	0.079	0.377
Systolic arterial pressure (mmHg)	-0.098	0.276
Diastolic arterial pressure (mmHg)	-0.007	0.936
Hemorrhage locations (lobar/deep)	0.024	0.793
Intraventricular hemorrhage	0.192	0.031
Subarachnoidal hemorrhage	0.157	0.080
NIHSS scores	0.606	< 0.001
Hematoma volume (ml)	0.670	< 0.001
Blood leucocyte count (×10^9^/l)	0.058	0.516
Blood glucose levels (mmol/l)	0.189	0.034

Bivariate correlations were showed as *ρ* values by Spearman’s correlation coefficient test

*NIHSS* indicates National Institutes of Health Stroke Scale

### Serum sestrin2 as a potential predictor of END after ICH

**Fig. 4:**
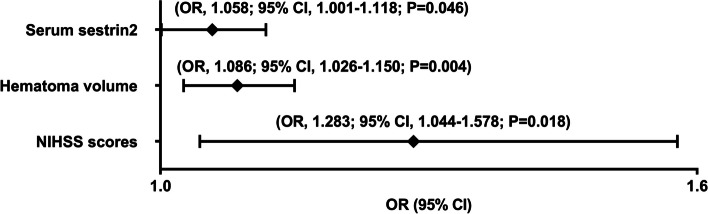
Forest plot showing multivariate logistic regression results of early neurologic deterioration after acute intracerebral hemorrhage.  Serum sestrin2 levels, National Institutes of Health Stroke Scale scores and hematoma volume were independently predictive of early neurologic deterioration following acute intracerebral hemorrhage (all *P*  < 0.05).  NIHSS indicates National Institutes of Health Stroke Scale; OR, odds ratio; 95% CI, 95% confidence interval

**Fig. 5:**
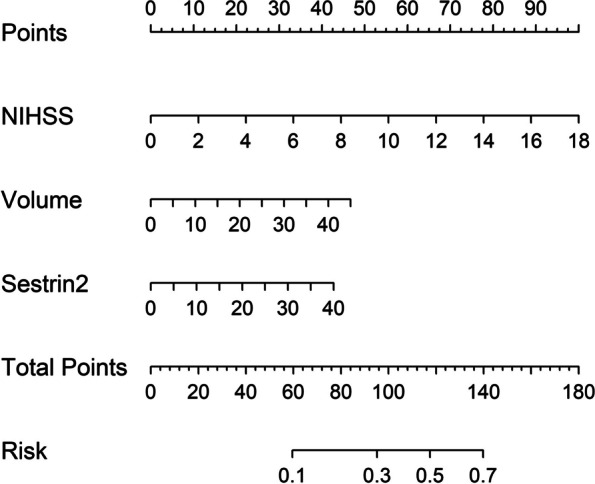
Nomogram exhibiting prediction model of early neurologic deterioration following acute intracerebral hemorrhage. Serum sestrin2 levels, National Institutes of Health Stroke Scale scores and hematoma volume were integrated to predict early neurologic deterioration after acute intracerebral hemorrhage. NIHSS indicates National Institutes of Health Stroke Scale.

**Fig. 6:**
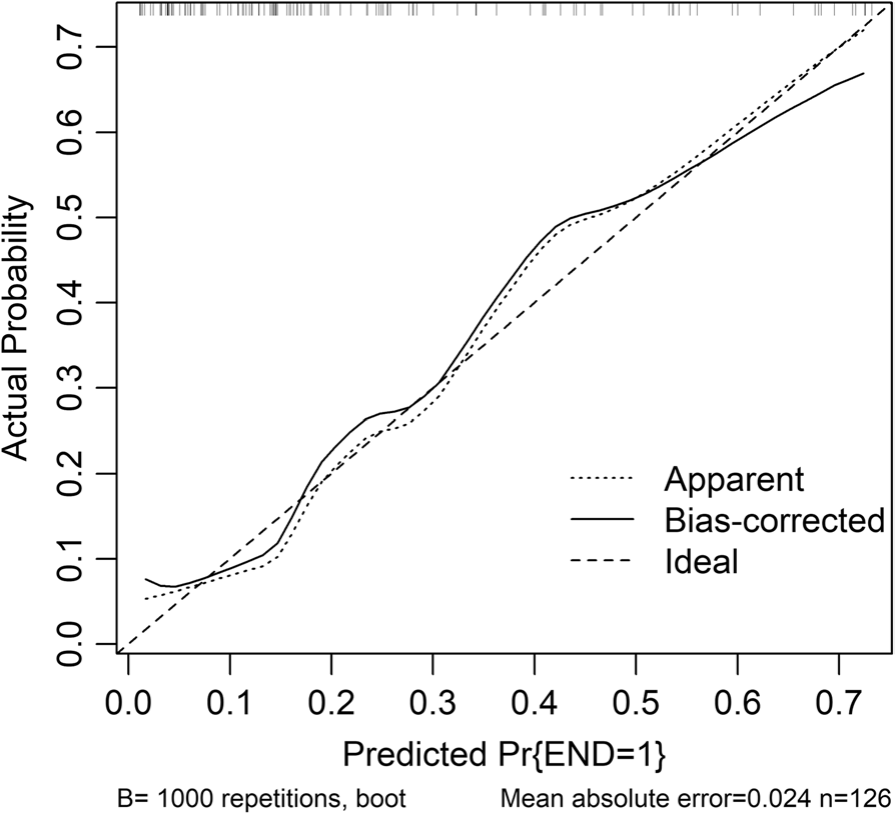
Calibration curve exhibiting stability of prediction model of early neurologic deterioration following acute intracerebral hemorrhage. Prediction model containing serum sestrin2 levels, National Institutes of Health Stroke Scale scores and hematoma volume relatively stably predicted early neurologic deterioration after acute intracerebral hemorrhage. END denotes early neurologic deterioration

**Fig. 7:**
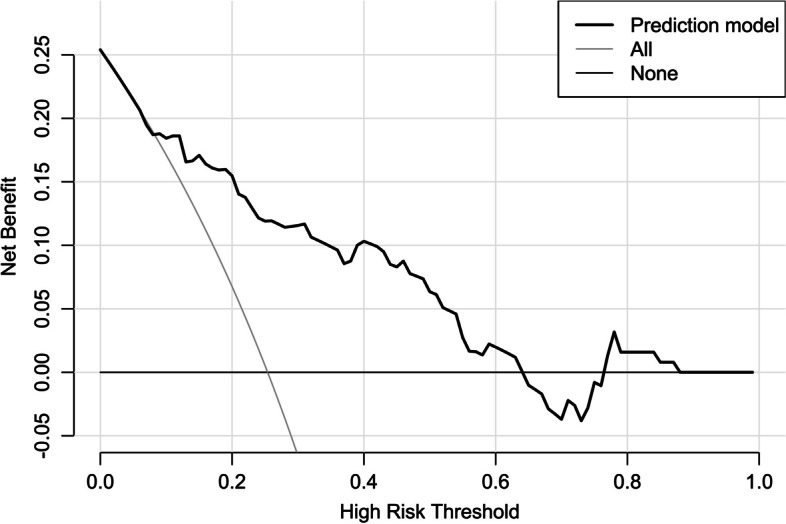
Decision curve showing clinical benefit of prediction model of early neurologic deterioration following acute intracerebral hemorrhage. Prediction model containing serum sestrin2 levels, National Institutes of Health Stroke Scale scores and hematoma volume was of clinical benefit for predicting early neurologic deterioration after acute intracerebral hemorrhage

**Fig. 8:**
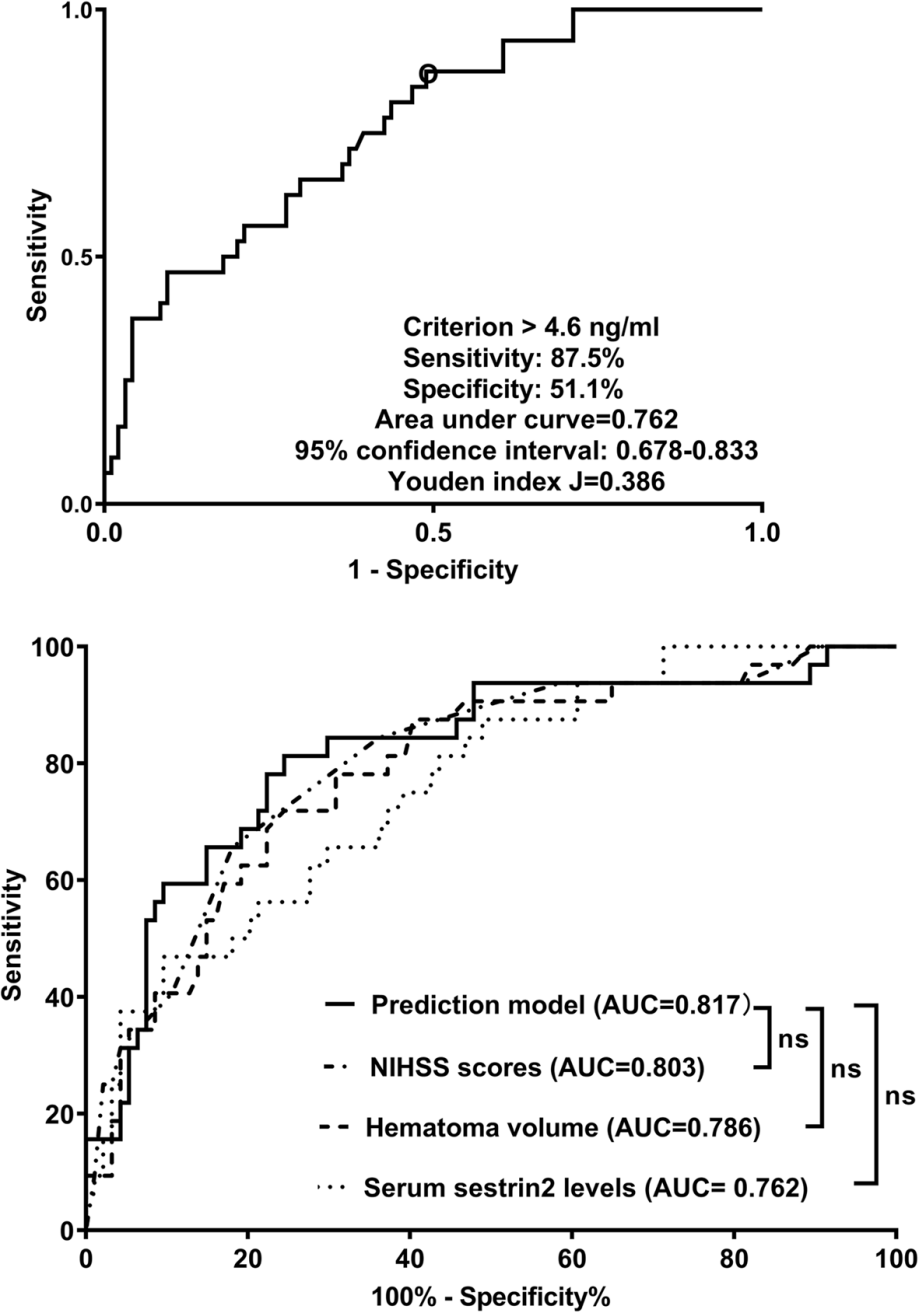
Receiver operating characteristic curve with respect to predictive value of serum sestrin2 levels for early neurologic deterioration after acute intracerebral hemorrhage. Serum sestrin2 levels significantly predicted early neurologic deterioration, and its predictive ability was comparable to those of National Institutes of Health Stroke Scale scores and hematoma volume (both *P*>0.05). However, the predictive ability of prediction model numerically exceeded any among National Institutes of Health Stroke Scale scores, hematoma volume and serum sestrin2 levels (all *P*>0.05). NIHSS indicates National Institutes of Health Stroke Scale; AUC, area under curve; ns, nonsignificant

Altogether, there were 32 patients with END in this cohort. In Table 3, as compared to patients without END, those with END had substantially elevated NIHSS scores, hematoma volumes, serum sestrin2 levels and blood glucose levels (all *P* < 0.05). The binary logistic regression analysis showed that NIHSS scores, hematoma volumes and serum sestrin2 levels were the independent predictors of END (all *P* < 0.05; Fig. 4). In Fig. 5, a nomogram, which contained NIHSS scores, hematoma volumes and serum sestrin2 levels, described END prediction model. Moreover, the prediction model was comparatively stable (Fig. 6) and also was relatively clinically beneficial (Fig. 7). Under the ROC curve (Fig. 8), serum sestrin2 levels were of discriminatory efficiency for predicting END and using Youden method, a best value of serum sestrin2 levels was identified; moreover, its predictive ability was comparable to those of NIHSS scores and hematoma volume (both *P* > 0.05). However, the predictive ability of prediction model numerically exceeded that of NIHSS scores, hematoma volume and serum sestrin2 levels alone (all *P* > 0.05).

**Table 3:** Factors related to early neurologic deterioration after acute intracerebral hemorrhage

Variables	Presence of early neurologic deterioration	Absence of early neurologic deterioration	*P* values
Number	32	94	
Age (years)	60.2 ± 10.6	62.5 ± 10.1	0.270
Gender (male/female)	17/15	52/42	0.829
Body mass index (kg/m^2^)	24.8 ± 2.9	24.0 ± 3.5	0.253
Hypertension	20 (62.5%)	55 (58.5%)	0.691
Diabetes mellitus	12 (37.5%)	19 (20.2%)	0.050
Hyperlipidemia	10 (31.3%)	30 (31.9%)	0.944
Coronary heart disease	3 (9.4%)	7 (7.5%)	0.727
Cigarette smoking	11(34.4%)	36 (38.3%)	0.692
Alcohol drinking	11 (34.4%)	38 (40.4%)	0.544
Statin use	8 (25.0%)	25 (26.6%)	0.859
Anticoagulant use	2 (6.3%)	5 (5.3%)	0.843
Antiplatelet use	6 (18.8%)	12 (12.8%)	0.403
Antihypertensive use	17 (53.1%)	50 (53.2%)	0.995
Hypoglycemic use	10 (31.3%)	15 (16.0%)	0.061
Admission time (h)	10.4 (7.6–15.5)	9.0 (4.9–11.9)	0.127
Sampling time (h)	11.7 (9.0-17.4)	10.6 (6.5–13.7)	0.157
Systolic arterial pressure (mmHg)	137.4 ± 22.9	144.7 ± 22.8	0.119
Diastolic arterial pressure (mmHg)	83.5 ± 7.5	86.5 ± 10.4	0.080
Hemorrhage locations (lobar/deep)	7/25	26/68	0.520
Intraventricular hemorrhage	10 (31.3%)	21 (22.3%)	0.312
Subarachnoidal hemorrhage	4 (12.5%)	7 (7.5%)	0.469
NIHSS scores	12 (11–15)	9 (6–11)	< 0.001
Hematoma volume (ml)	22 (15–29)	11 (8–16)	< 0.001
Blood leucocyte count (×10^9^/l)	8.7 (6.2–11.0)	7.8 (6.3–9.6)	0.368
Blood glucose levels (mmol/l)	13.3 (10.1–18.5)	10.8 (9.1–12.4)	0.010
Serum sestrin2 levels (ng/ml)	14.0 (5.8–24.0)	4.6 (2.4–12.4)	< 0.001

Data were reported as count (percentage), mean ± standard deviation or median (upper-lower quartile) based on datum patterns. Statistical methods included the Chi-square test, Fisher exact test, Student’s 𝑡-test and Mann-Whitney test

*NIHSS indicates *National Institutes of Health Stroke Scale

### Serum sestrin2 as a potential predictor of poor prognosis at six months after ICH

**Fig. 9:**
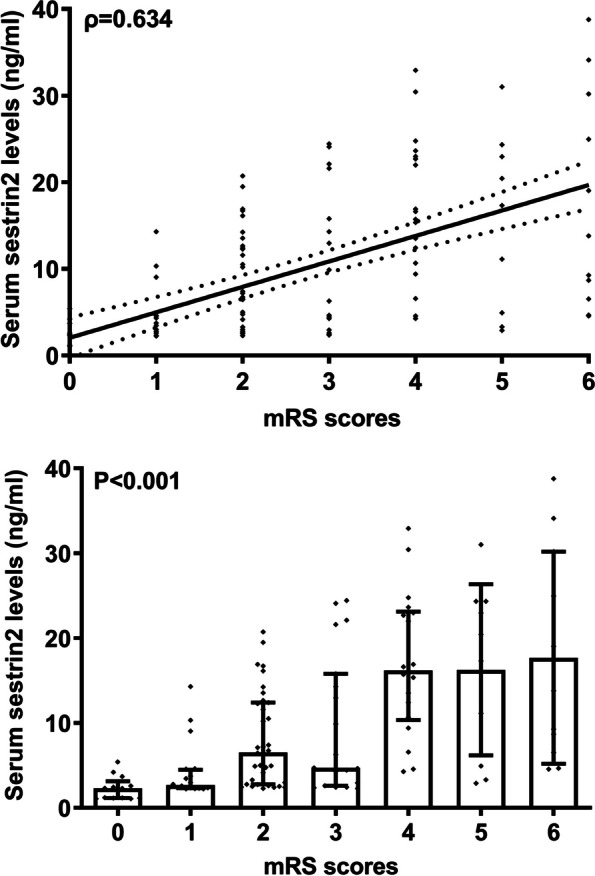
Graph displaying relationship between serum sestrin2 levels and modified Rankin Scale scores at six months after acute intracerebral hemorrhage. Serum sestrin2 levels were tightly correlated with modified Rankin Scale scores (*P*<0.001) and were significantly lowest in patients with score 0, followed by scores 1-5, and were substantially highest in those with score 6 (*P*<0.001). mRS indicates modified Rankin Scale

**Fig. 10:**
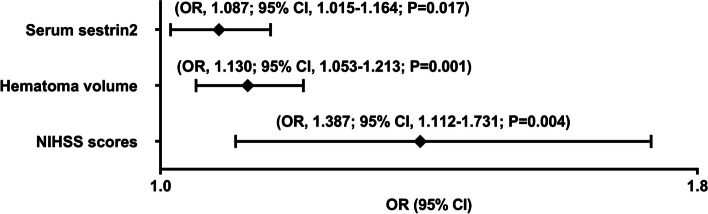
Forest plot showing multivariate logistic regression results of poor prognosis at six months after acute intracerebral hemorrhage. Serum sestrin2 levels, National Institutes of Health Stroke Scale scores and hematoma volume were independently associated with six-month poor prognosis following acute intracerebral hemorrhage (all *P*<0.05). NIHSS indicates National Institutes of Health Stroke Scale; OR, odds ratio; 95% CI, 95% confidence interval

**Fig. 11:**
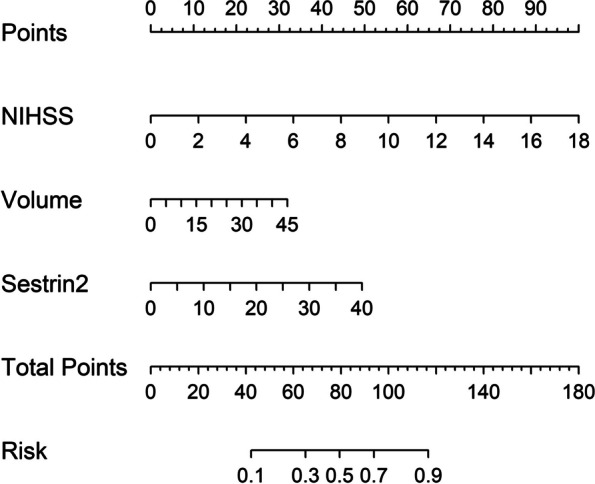
Nomogram exhibiting prediction model of six-month poor prognosis following acute intracerebral hemorrhage. Serum sestrin2 levels, National Institutes of Health Stroke Scale scores and hematoma volume were combined to predict six-month poor prognosis after acute intracerebral hemorrhage. NIHSS indicates National Institutes of Health Stroke Scale

**Fig. 12:**
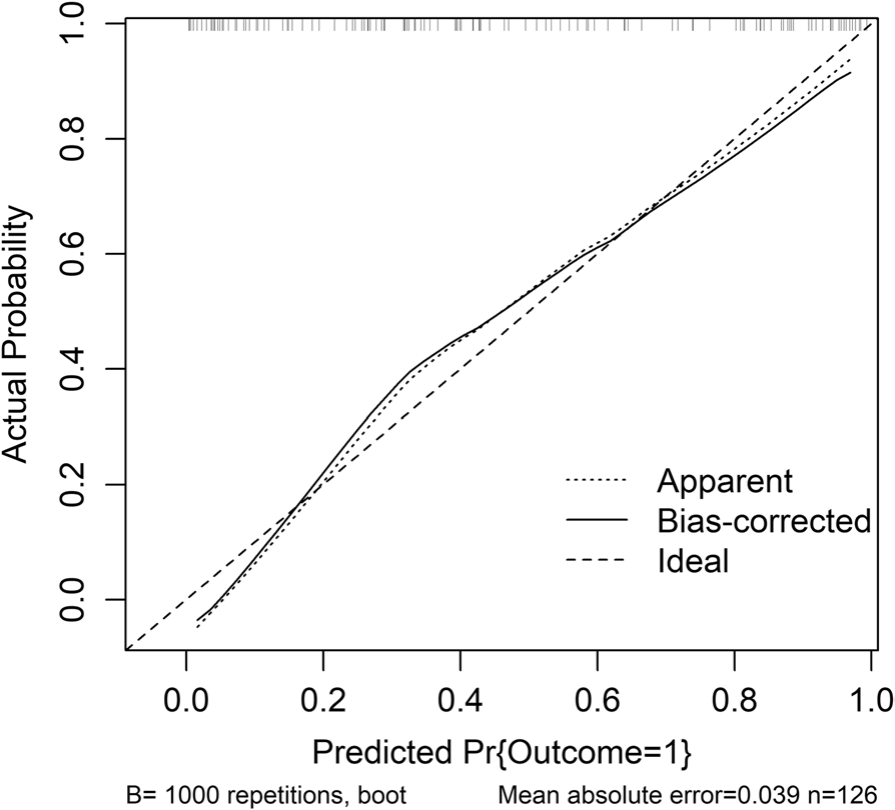
Calibration curve exhibiting stability of prediction model of six-month poor prognosis following acute intracerebral hemorrhage. Prediction model containing serum sestrin2 levels, National Institutes of Health Stroke Scale scores and hematoma volume was relatively stable for predicting six-month poor prognosis after acute intracerebral hemorrhage

**Fig. 13:**
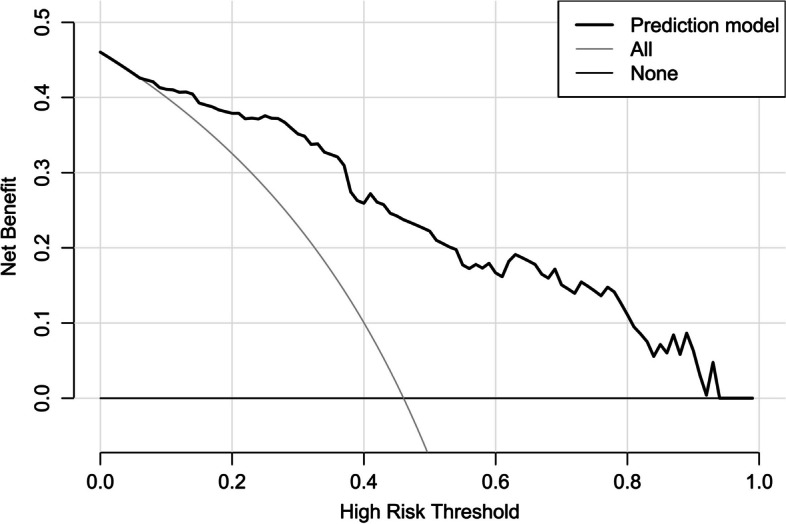
Decision curve showing clinical benefit of prediction model of six-month poor prognosis following acute intracerebral hemorrhage. Prediction model containing serum sestrin2 levels, National Institutes of Health Stroke Scale scores and hematoma volume was clinically beneficial for predicting six-month poor prognosis after acute intracerebral hemorrhage

**Fig. 14:**
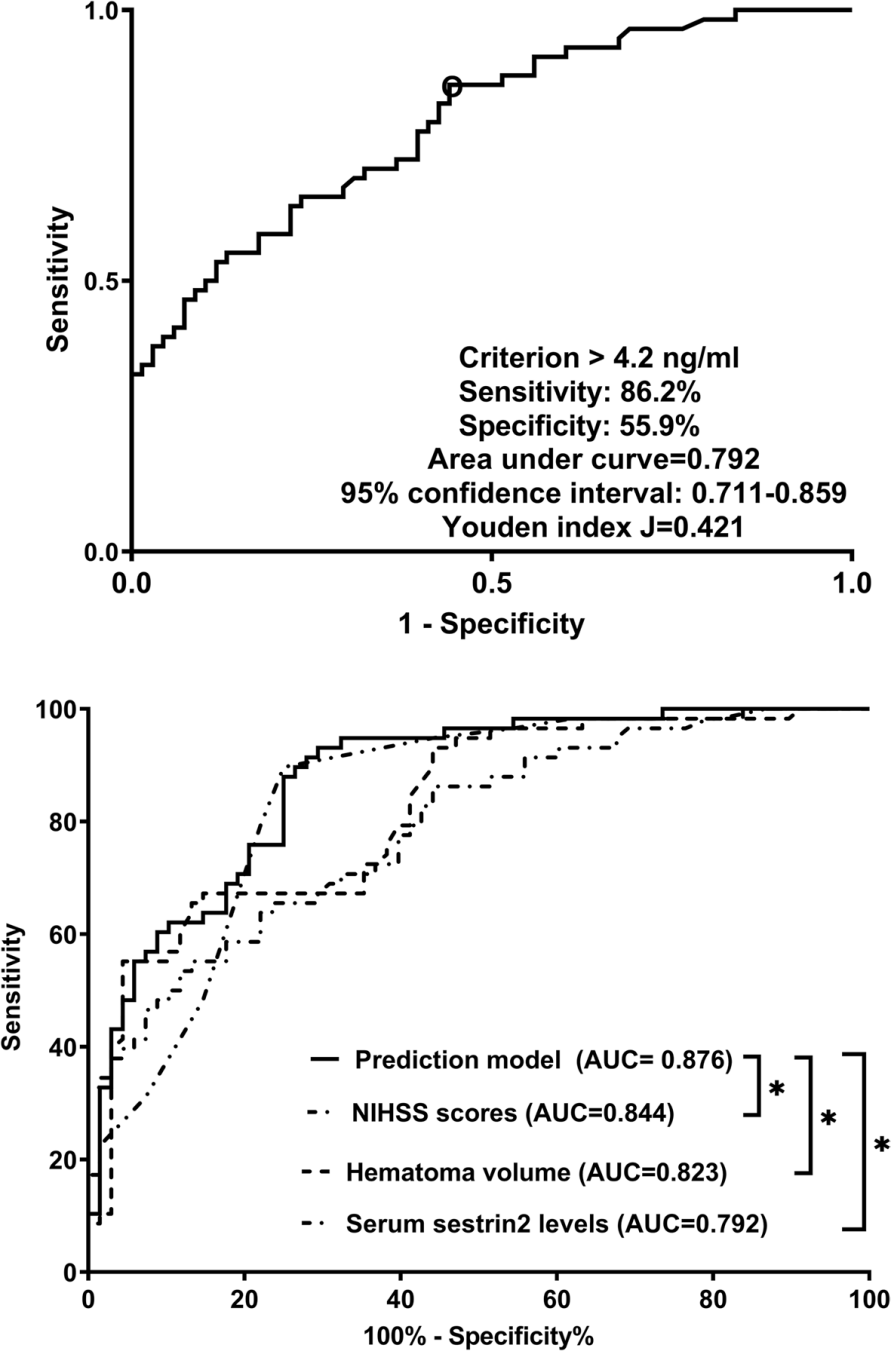
Receiver operating characteristic curve with respect to predictive ability of serum sestrin2 levels for six-month poor prognosis after acute intracerebral hemorrhage. Serum sestrin2 levels significantly discriminated patients at risk of six-month poor prognosis, and its predictive capability was similar to those of National Institutes of Health Stroke Scale scores and hematoma volume (both *P*>0.05). Moreover, the discriminative ability of prediction model was significantly higher than any among National Institutes of Health Stroke Scale scores, hematoma volume and serum sestrin2 levels (all *P*<0.05). NIHSS indicates National Institutes of Health Stroke Scale; AUC, area under curve. ^*^P<0.05

Poststroke six-month mRS scores ranged from 0 to 6, with a median value of 2 (percentiles 25th – 75th, 1–4). In addition, mRS scores from 0 to 6 were revealed in 13, 20, 35, 19, 18, 10 and 11 patients respectively. As displayed in Fig. 9, serum sestrin2 levels were markedly positively correlated with mRS scores (*P* < 0.001) and were significantly increased in the order of mRS scores from 0 to 6 (*P* < 0.001). In total, 58 ICH patients suffered from a poor prognosis following stroke. Admittedly, serum sestrin2 levels were pronouncedly higher in patients with poor prognosis than in those with good prognosis (*P* < 0.001; Table 3). Also, as compared with patients presenting with good prognosis, those with development of poor prognosis had significantly elevated NIHSS scores, hematoma volume and blood glucose levels, as well as displayed substantially increased percentages of intraventricular hemorrhage and subarachnoid hemorrhage (all *P* < 0.05; Table 4). Multivariate analysis showed that serum sestrin3 levels, NIHSS scores and hematoma volume independently predicted poor prognosis after ICH (all *P* < 0.05; Fig. 10). A nomogram, in which the preceding three independent predictors were integrated, was configured to delineate the prediction model (Fig. 11). Also, the prediction model was relatively reliable (Fig. 12) and was in possession of clinical value (Fig. 13). Under the ROC curve (Fig. 14), serum sestrin2 levels significantly predicted poor prognosis (*P* < 0.001). Using the Youden method, its optimal value was selected for prognostic prediction. Moreover, its predictive capability was in range of NIHSS scores and hematoma volume (both *P* > 0.05; Fig. 14). Of note, the prognostic predictive ability of prediction model was dramatically higher than that of serum sestrin2 levels, NIHSS scores and hematoma volume alone (all *P* < 0.05; Fig. 14).

**Table 4:** Factors related to six-month poor prognosis after acute intracerebral hemorrhage

Variables	mRS 3–6	mRS 0–2	*P* value
Number	58	68	
Age (years)	62.1 ± 9.8	61.7 ± 10.6	0.822
Gender (male/female)	28/30	41/27	0.177
Body mass index (kg/m^2^)	24.1 ± 3.1	24.3 ± 3.6	0.792
Hypertension	37 (63.8%)	38 (55.9%)	0.367
Diabetes mellitus	14 (24.1%)	17 (25.0%)	0.911
Hyperlipidemia	19 (32.8%)	21 (30.9%)	0.822
Coronary heart disease	7 (12.1%)	3 (4.4%)	0.113
Cigarette smoking	18 (31.0%)	29 (42.7%)	0.179
Alcohol drinking	19 (32.8%)	30 (44.1%)	0.192
Statin use	16 (27.6%)	17 (25.0%)	0.742
Anticoagulant use	3 (5.2%)	4 (5.9%)	0.862
Antiplatelet use	6 (10.3%)	12 (17.7%)	0.243
Antihypertensive use	32 (55.2%)	35 (51.5%)	0.678
Hypoglycemic use	12 (20.7%)	13 (19.1%)	0.825
Admission time (h)	9.2 (6.8–12.4)	8.9 (4.8–14.7)	0.862
Sampling time (h)	10.9 (8.3–14.6)	10.4 (6.5–16.4)	0.818
Systolic arterial pressure (mmHg)	140.3 ± 23.2	145.1 ± 22.8	0.243
Diastolic arterial pressure (mmHg)	85.3 ± 10.0	86.1 ± 9.6	0.638
Hemorrhage locations (lobar/deep)	17/41	16/52	0.462
Intraventricular hemorrhage	20 (34.5%)	11 (16.2%)	0.017
Subarachnoidal hemorrhage	9 (15.5%)	2 (2.9%)	0.013
NIHSS scores	11 (11–13)	8 (4–10)	< 0.001
Hematoma volume (ml)	21 (13–28)	9 (6–15)	< 0.001
Blood leucocyte count (×10^9^/l)	8.3 (6.2–10.9)	7.9 (6.3–9.4)	0.829
Blood glucose levels (mmol/l)	12.9 (9.5–17.1)	10.8 (9.2–12.3)	0.014
Serum sestrin2 levels (ng/ml)	13.7 (4.7–23.0)	3.6 (2.4–7.3)	< 0.001

Data were summarized in form of number (proportion), mean ± standard deviation or median (upper-lower quartile) based on datum patterns. The Chi-square test, Fisher exact test, Student’s 𝑡-test and Mann-Whitney test were employed for statistical analysis

*NIHSS* denotes National Institutes of Health Stroke Scale, *mRS *modified Rankin Scale

## Discussion

To the best of our knowledge, this is a first series for investigating serum sestrin2 levels after ICH. Our main findings were that: (1) serum sestrin2 levels were profoundly raised in humans with ICH, as opposed to healthy controls; (2) serum sestrin2 levels at baseline after ICH were independently correlated with admission NIHSS scores and baseline hematoma volumes; (3) Serum sestrin2 levels independently predicted END after ICH, with efficient discriminative ability under the ROC curve; (4) whether mRS was identified as a continuous or categorical variable, or mRS was dichotomized; or whether univariate analysis or multivariate analysis was used; serum sestrin2 levels were confirmed to be closely associated with poor functional outcome indicated by mRS scores, with significant discriminative capability; (5) the prediction model was effective in predicting END and poor prognosis using multiple tests; moreover, the prognostic prediction model displayed higher discriminative efficiency, as compared to NIHSS scores and hematoma volume. The preceding data are strongly supportive of the assumption that serum sestrin2 may aid in severity assessment and prognostic prediction of acute ICH.

Up to now, compelling evidence has indicated that sestrin2 may be a protective factor in acute brain injury diseases. Specifically, in rats with ischemic stroke induced using a photothrombotic procedure, it was demonstrated via techniques of sestrin2 overexpression and silencing that sestrin2 may profoundly promote angiogenesis in cerebral tissues [22]. Similarly, recombinant human sestrin2 pronouncedly decreased cerebral infarct and brain edema, while silencing sestrin2 markedly generated opposite effects after severe hypoxic-ischemic injury in neonatal rats [13]. Also, in mice with transient middle cerebral artery occlusion, neurological deficits, infarction volume and cell apoptosis were dramatically attenuated by exogenous sestrin2; and condition medium from BV2 cells cultured with sestrin2 significantly reduced neuronal apoptosis after oxygen-glucose deprivation in vitro [23]. Another experiment showed that sestrin2 overexpression in the brain substantially decreased oxidative stress, neurological deficit, brain edema and neuronal apoptosis of mice with traumatic brain injury [14]. In addition, exogenous recombinant human sestrin2 significantly lessened neuroinflammatory insults and oxidative stress, and improved neurologic function of mice after subarachnoid hemorrhage; moreover, recombinant human sestrin2 substantially enhanced M2-like microglia polarization and depressed the number of M1-like microglia after subarachnoid hemorrhage [15]. Thus, sestrin2 may actually play a brain protective role after acute brain injury.

After experimental traumatic brain injury or acute cerebral ischemia, sestrin2 expressions were obviously up-regulated in animal cerebral cortex [13, 14]. Alternatively, sestrin2 was localized in neurons and microglia in brain of mice with subarachnoid hemorrhage [15]. There was a significant elevation of serum sestrin2 levels in patients with Parkinson’s disease and Alzheimer’s disease or mild cognitive impairment [16, 17]. Our study showed that serum sestrin2 levels were substantially elevated after acute ICH. Given the protective effects of sestrin2 [13–15, 22, 23], an elevation of sestrin2 levels is likely to be a compensatory response to acute brain injury. On the one hand, oxidative stress may be the most important reason for sestrin2 secretion, thereby leading to sestrin2 compensatory increase in oxidative stress environment [24, 25]. On the other hand, increased sestrin2 levels may not be sufficient to counter dominant oxidative stress [24, 25], therefore resulting in a false impression that higher serum sestrin levels may lead to poorer clinical outcomes in patients with ICH.

We found significantly increased serum sestrin2 levels after ICH and further investigated relationship between serum sestrin2 levels and illness severity in addition to END and long-term poor prognosis following ICH. On the one hand, serum sestrin2 levels were confirmed to be independently correlated with NIHSS scores and hematoma volumes. On the other hand, serum sestrin2 levels were positively correlated with mRS scores and were significantly elevated in order of mRS scores, which was identified as a categorical variable. Moreover, mRS scores were dichotomized and therefore multivariate model was established. Afterwards, serum sestrin2 levels were found to be independently associated with poor prognosis and END. Using ROC curve analysis, prediction model containing serum sestrin2 levels showed higher advantage in predicting poor prognosis, but not in predicting END. In addition, the prediction model displayed high clinical benefit using decision curve analysis and exhibited reliable discriminative ability under calibration curve. Overall, serum sestrin is likely to represent a potential biomarker, whose measurement may be of clinical significance in severity assessment and prognostic prediction of ICH.

## Conclusions

To the best of our knowledge, there is a paucity of data available regarding serum sestrin2 levels after acute brain injury. It is found that serum sestrin2 levels are significantly raised after acute ICH, and the levels are highly correlated with stroke severity and are markedly associated with END and long-term neurologic functional outcome. The results of the current study indicate a significant potential of serum sestrin2 in the prognosis prediction and severity assessment of ICH, and such a serum sestrin2 identification may be essential in clinical work of ICH.

## Data Availability

The datasets generated and/or analyzed during the current study are not publicly available due for they are personal data but are available from the corresponding author on reasonable request.
